# State-Dependent Modulation of Visual Evoked Potentials in a Rodent Genetic Model of Electroencephalographic Instability

**DOI:** 10.3389/fnsys.2018.00036

**Published:** 2018-08-15

**Authors:** Janne Grønli, Michelle A. Schmidt, Jonathan P. Wisor

**Affiliations:** ^1^Department of Biological and Medical Psychology, University of Bergen, Bergen, Norway; ^2^Department of Biomedical Sciences, Elson S. Floyd College of Medicine, Washington State University, Spokane, WA, United States; ^3^Sleep and Performance Research Center, Washington State University, Spokane, WA, United States

**Keywords:** EGR3, quiet wakefulness, beta frequency, electroencephalogram, sleep, arousal, 5HT2A, visual evoked potential (VEP)

## Abstract

Despite normal sleep timing and duration, *Egr3*-deficient (*Egr3*^−/−^) mice exhibit electroencephalographic (EEG) characteristics of reduced arousal, including elevated slow wave (1–4 Hz) activity during wakefulness. Here we show that these mice exhibit state-dependent instability in the EEG. Intermittent surges in EEG power were found in *Egr3*^−/−^ mice during wakefulness and rapid eye movement sleep, most prominently in the beta (15–35 Hz) range compared to wild type (*Egr3*^+/+^) mice. Such surges did not coincide with sleep onset, as the surges were not associated with cessation of electromyographic tone. Cortical processing of sensory information by visual evoked responses (VEP) were found to vary as a function of vigilance state, being of higher magnitude during slow wave sleep (SWS) than wakefulness and rapid eye movement sleep. VEP responses were significantly larger during quiet wakefulness than active wakefulness, in both *Egr3*^−/−^ mice and *Egr3*^+/+^ mice. EEG synchronization in the beta range, previously linked to the accumulation of sleep need over time, predicted VEP magnitude. *Egr3*^−/−^ mice not only displayed elevated beta activity, but in quiet wake, this elevated beta activity coincides with an elevated evoked response similar to that of animals in SWS. These data confirm that (a) VEPs vary as a function of vigilance state, and (b) beta activity in the EEG is a predictor of state-dependent modulation of visual information processing. The phenotype of *Egr3*^−/−^ mice indicates that *Egr3* is a genetic regulator of these phenomena.

## Introduction

Sensory evoked potentials are a rich source of information for quantifying the processing of sensory information by the central nervous system. Evoked potentials can be detected in sleep as well as during wakefulness, however profound modifications in the shape and timing of evoked potentials are found to occur across states (reviewed in Colrain and Campbell, 2007; Rector et al., 2009).

State-dependent modulation of evoked potentials has been demonstrated in humans (Weitzman and Kremen, 1965) and rodents (Rector et al., 2005, 2009; Phillips et al., 2011). During slow wave sleep (SWS) there is an elevated magnitude of sensory evoked potentials relative to wakefulness. Deep SWS exhibits a further elevated magnitude of evoked potential relative to light SWS characterized by synchronized burst firing of large number of cortical cells (Nielsen-Bohlman et al., 1991). This form of modulation is specific to SWS (Nielsen-Bohlman et al., 1991). Stimuli administered during rapid eye movement sleep (REMS) yield evoked potential components similar to those related to active attention during wake (Colrain and Campbell, 2007). Evoked potentials can thus be used to gauge cortical vigilance (or reduced arousal). Moreover, some studies have described state-specific modulation of somatosensory (Crochet and Petersen, 2006) or visual (Niell and Stryker, 2010) inputs to cerebral cortex depending on the state of wakefulness (active wake, AW vs. quiet wake, QW). During QW, slow oscillations in the local field potential (LFP) can be detected in individual barrels of the primary somatosensory cortex while other somatosensory barrels continue to exhibit wake-like LFPs (Rector et al., 2009). These local SWS states in the rodent barrel cortex have been important to our understanding of state-dependent modulation of somatosensory information processing. Whisker deflection during the local SWS state induces SWS-like high magnitude VEPs in the corresponding barrel of the somatosensory cortex (Rector et al., 2009). These SWS-like VEPs have been shown to coincide temporally with errors in the performance of tasks in response to stimulation of the whisker that drives that barrel (Krueger et al., 2013). Such observations demonstrate that cortical connectivity is disrupted at the local circuit level as a consequence of local SWS-like events. However, the neurobiological underpinnings of this local sleep state still remain uncertain.

In the cerebral cortex, experience-dependent plastic processes are influenced by vigilance state-dependent expression of plasticity-regulatory genes (Seibt et al., 2012). These processes are contingent on changes in cellular second messenger pathways that are, in turn, dependent on vigilance states (Dumoulin et al., 2015). One plasticity-regulatory gene that is upregulated as a consequence of sleep deprivation is the immediate early gene early growth response 3 (*Egr3*) (Terao et al., 2006). Mice deficient for *Egr3 (Egr3*^−/−^*)* show normal sleep timing and duration, with the exception of a modest reduction in REMS. However, during QW, *Egr3*^−/−^ mice exhibit elevated electroencephalographic (EEG) synchrony in the 3–8 Hz range and 15–35 Hz (beta) range relative to *Egr3*^+/+^ mice (Grønli et al., 2016a). This elevated synchrony during QW may indicate reduced sensory processing capabilities in the absence of a functioning *Egr3* locus. One purpose of the current study was to determine whether elevated EEG synchrony during QW coincides with alterations in the VEPs of *Egr3*^−/−^ mice relative to *Egr3*^+/+^ mice.

*Egr3* is associated with schizophrenia risk (Yamada et al., 2007). A reduced expression of *Egr3* is reported in the brains of schizophrenics (Mexal et al., 2005; Yamada et al., 2007) and an allelic variant of the *Egr3* locus confers increased likelihood of schizophrenia (albeit in an ethnicity-dependent-manner) (Huentelman et al., 2015). Patients with schizophrenia demonstrate significant impairments of sensory processing (Lebedeva et al., 2003; Schechter et al., 2005; Brockhaus-Dumke et al., 2008). Changes in sensory evoked potentials in schizophrenics relative to non-schizophrenic subjects parallel the changes that occur within the normal population under experimentally controlled sleep deprivation. It has been proposed that deficits in cortical arousal mechanisms underlie evoked potential abnormalities in schizophrenia (Ettinger and Kumari, 2015). While the understanding of sensory deficits in schizophrenia is still incomplete, research appears to suggest that deficits in visual processing occur as a consequence of a distributed impairment involving many cortical areas and their connectivity (Uhlhaas and Singer, 2010; Lalor et al., 2012). *Egr3*^−/−^ mice exhibit schizophrenia-like behavioral phenotypes, including failure to habituate to novel social stimuli and acoustic stimuli (Gallitano-Mendel et al., 2007). Hence, studies on sensory processing in *Egr3*^−/−^ mice may yield insights into the influence of arousal mechanisms and deficits in experience-dependent plastic processes in schizophrenia.

In the present study, VEPs in visual cortex (V1) were found to vary as a function of vigilance state, being of higher magnitude during SWS than wakefulness and REMS. In addition, QW was characterized by higher VEP magnitude relative to AW. These findings were found independently of genotype. *Egr3*^−/−^ mice exhibited intermittent surges in beta (15–35 Hz) activity similar to those that occur under high sleep pressure in enforced wakefulness in wild type mice (Grønli et al., 2016b). In parallel with this EEG instability phenotype, *Egr3*^−/−^ mice also exhibited state-dependent (QW-specific) elevation of VEP magnitude during wakefulness, complementing their tonic EEG phenotype. Independently of genotype, EEG synchronization in the beta range predicted VEP magnitude. These data demonstrate the utility of EEG activity in the beta range as a predictor of state modulation of cortical sensory processing, and suggest a role for *Egr3* in regulating state-dependent changes in sensory processing.

## Materials and methods

### Use of animals in research

This study was approved by the institutional animal care and use committee of Washington State University (Protocol Number: 3932) and conducted in accordance with National Research Council guidelines and regulations controlling experiments in live animals (NRC, 1996).

### Animals

The line of *Egr3*^−/−^ mice used in the current work were generated (Tourtellotte and Milbrandt, 1998), behaviorally phenotyped (Gallitano-Mendel et al., 2007), and polysomnographically phenotyped (Grønli et al., 2016a) in previous publications. Detailed characterization of breeding and genotyping of the colony at Washington State University is reported elsewhere (Grønli et al., 2016a). All animals were housed in a 12 h light/12 h dark cycle, (except during VEP measurement; see *Experimental protocol: visual evoked potentials*) with food and water available *ad libitum*.

### Experimental protocol: baseline EEG phenotypes

Mice of both sexes (15 *Egr3*^+/+^, 9 female and 15 *Egr3*^−/−^, 9 female) were used. Mice weighing between 20 and 24 grams (*Egr3*^+/+^ 7–15 weeks of age, *Egr3*^−/−^ 10–20 weeks of age) were subjected to surgical implantation of bilateral neck electromyographic (EMG) and fronto-parietal EEG electrodes under anesthesia of isoflurane (5% induction; 1–3% to maintain 0.5–1 Hz respiration rate). Relative to bregma, the EEG electrodes were placed AP = 1.0 mm, ML = ±1.5 mm and AP = −3.0 mm, ML = +1.5 mm for reference electrode. Wires from EEG and EMG leads were then soldered to a plastic headmount connector affixed to the skull (Pinnacle Technologies, part # 8201-SS). Analgesic (Buprenorphine; 0.1 mg/kg, s.c.) and anti-inflammatory (flunixin meglumine; 0.1 mg/kg, s.c.) agents were given 2 days post-surgery. At least 2 weeks were allowed following surgery for recovery (see Wisor et al., 2011; Clegern et al., 2012) for more details of the surgical procedures.

Undisturbed 24 h baseline EEG/EMG were collected at 400 Hz starting at lights-on (Zeitgeber Time, ZT0) after one night of acclimatization to the cylindrical recording cage (diameter 25 cm × height 20 cm). Significant sex differences in the phenotypes described in the current study were not detected and are not further considered. Data on sleep timing and the vigilance state-specific EEG characteristics are reported in Grønli et al. (2016a).

### Experimental protocol: visual evoked potentials

VEPs were measured in a distinct cohort of mice from the one used for baseline EEG phenotypes described above. Body-weight matched male mice (4 male *Egr3*^+/+^ and 4 male *Egr3*^−/−^*)*, weighing between 20 and 24 grams were used. Age ranges in days at the time of recording onset were 75–91 days for *Egr3*^+/+^ and 120–135 for *Egr3*^−/−^. The mice were subjected to surgical implantation of EMG and fronto-occipital EEG electrodes under the surgical protocol described above. A stainless-steel polyimide-insulated EEG electrode (Plastics One part #E363/1/SPC; diameter: 0.280 mm) was implanted in V1 visual cortex at AP = −3.0 mm, and ML = +2.5 mm relative to bregma, and at 0.5 mm depth from the skull. Reference, AP = +2.5 mm and ML = −1.5 mm, and ground electrodes AP = +2.5 mm and ML = +1.5 mm were implanted in the frontal cortex. At least 2 weeks were allowed following surgery for recovery.

**Figure 1:**
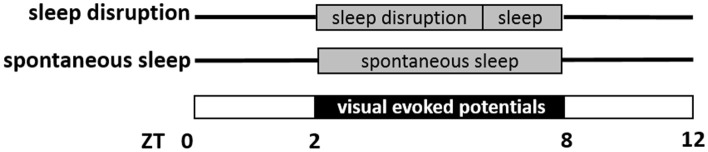
Schematic representation of visual evoked potential (VEP) data collection. Mice were studied on two separate days, a sleep disruption day and a spontaneous sleep day. On both days, mice were in a skeleton photoperiod, in which lights were on from Zeitgeber Time ZT0 to ZT2 and again ZT8 to ZT12. In the interim from ZT2 to ZT8, VEPs were measured in response to light pulses (10 ms duration, frequency 1 Hz) administered in a background of darkness. On the sleep disruption day, mice were subjected to sleep disruption (ZT2–ZT6) and then allowed to sleep spontaneously (ZT6–ZT8). On the spontaneous sleep day, mice were allowed to sleep spontaneously from ZT2 to ZT8.

Sampling of EEG and EMG signals started after one night of acclimatization to the cylindrical recording cage. Data from two 6-hr sessions were recorded, starting at ZT2 and separated by 7 days. As schematized in Figure 1, mice were subjected to a skeleton photoperiod on each of the two recording days, with lights-on from ZT0 to ZT2, lights-off from ZT2 to ZT8 (for presentation of visual stimuli in a background of darkness) and lights-on again from ZT8 to ZT12. It was necessary to place animals in constant darkness at this time, so that VEPs evoked by the LED could be measured against a background of no visual stimulation. From a circadian standpoint, such a “skeleton photoperiod” does not shift the circadian clock (Pittendrigh and Daan, 1976), as light from ZT0-ZT2 and ZT8-ZT12 maintains entrainment. And although darkness may transiently increase time awake through masking, it does not eliminate sleep, or sleep homeostasis (Mistlberger et al., 1983). The first recording session consisted of sleep disruption (SD) during the first 4 h of recording (ZT2–ZT6), and spontaneous sleep thereafter (ZT6–ZT8). SD was enforced by a rotating bar in the base of the cage disturbing the animals every 5–6 s (Wisor et al., 2011). The second session consisted of undisturbed sleep throughout the 6 h recording. The order of the two conditions was counterbalanced within each genotype. In both sessions, mice were subjected to a series of visual stimuli of 10 ms duration delivered at 1 Hz throughout the duration of the 6 h recording. The stimulus (465 nm wavelength) was generated by a light-emitting diode and delivered to the visual field via the end of a fiber optic cable affixed between the eyes. The fiber optic cable was attached to the EEG/EMG cable, and therefore did not require head restraint or prevent locomotion. The timing of each light pulse was monitored via a transistor-transistor logic (TTL) signal and recorded at 400 Hz as a binary trace in the file containing the EEG/EMG potentials.

### Vigilance state classification

In both experiments, the vigilance states of wakefulness, SWS and REMS were classified in 10 s epochs by an experienced scorer blind to the experimental conditions. Waking state epochs were further sub-classified as either QW (those epochs in which the root mean square of the EMG signal in the epoch was ≤33rd percentile of root mean square values across all wake epochs in the recording), or active wakefulness (AW; those epochs in which the root mean square of the EMG signal in the epoch was ≥66th percentile of root mean square values across all wake epochs in the recording). All other epochs of wake (EMG root mean square >33rd percentile and <66th percentile) were classified as intermediate wakefulness (IW). These criteria have been shown to be sufficient to discriminate epochs with high locomotor activity (AW) from epochs of behavioral quiescence (QW) (Grønli et al., 2016b).

### EEG state stability analysis

EEG state stability and VEP measurements were conducted separately within EMG-defined AW, IW, and QW, as both the EEG manifestation of accumulated sleep need in wild type mice (Grønli et al., 2016b), and the previously-reported EEG phenotype of *Egr3*^−/−^ mice (Grønli et al., 2016a), are detected only during QW and absent in AW.

To quantify the stability of EEG network activity within each vigilance state, we measured epoch-by-epoch changes of EEG power only when two consecutive epochs were of the same state classification (AW, IW, QW, SWS, or REMS). EEG power derived from fast Fourier transform (FFT) was calculated for each epoch by averaging EEG power within each of five 2-sec intervals encompassing the 10 s epoch (Grønli et al., 2016a). Power was summed within the delta (1–4 Hz), theta (5–8 Hz), alpha (9–12 Hz), and beta (15–35 Hz) ranges, all of which are potentially of interest as biomarkers for sleep need, as they vary as a function of time spent awake and waking substate (AW vs. QW; Grønli et al., 2016b). For each spectral band (delta, theta, alpha, beta) we derived two measures of state stability across consecutive epochs within each vigilance state: *the mean change*, and *the 90th percentile value* for change in spectral power across consecutive epochs of that state. To control for individual differences in total EEG power, the values were normalized as a percent change relative to total spectral power in that specific band in the first of the two epochs of uniform state across which the change in EEG power was measured.

### Visual evoked potential data analysis

To measure VEP waveform and magnitude the electrical potential was measured from the V1 visual cortex depth electrode at 400 Hz. The window analyzed was from 0.5 s before and to 0.5 s after stimulus onset, for every 10 ms visual stimulus delivered. These peri-stimulus histograms were sorted based on the vigilance state scored in the mice (AW, IW, QW, SWS, REMS) at stimulus onset. A state-specific average was generated by summation of all individual peri-stimulus histograms across the recording. VEP magnitude was measured by subtracting the (negative) potential in V1 cortex 150 ms after stimulus onset from the (positive) potential in V1 cortex 250 ms after stimulus onset in these average peri-stimulus curves, per previously published work on VEPs in the mouse (Sawtell et al., 2003; Frenkel and Bear, 2004). The resulting single state-specific value from each animal for each vigilance state was used as an input to the analysis of variance (ANOVA).

Beta activity in the EEG of wild type mice reflects distinct processes in AW and QW. Beta activity occurs in association with sensory processing in AW, but not in QW (Grønli et al., 2016b). In QW but not AW, beta activity increases as a function of time spent awake, independently of sensory input. To measure the relationship between beta activity and VEP waveforms, epochs of AW and QW were further segregated into two classes: low beta activity (≤20th percentile of that state within that recording), and high beta activity (≥80th percentile of that state within that recording). Average peri-stimulus histograms and VEP magnitude were generated for each of the two resulting substates (low beta/high beta) within each waking substate (AW vs. QW) in that animal's recording.

Measurement of EEG power spectra, EMG root mean square, and discrimination of states based on these metrics were performed with custom algorithms in the MATLAB programming language, which will be made available on request. All statistics were performed with Statistica version 13.2.

## Results

### Instability in EEG network activity in *Egr3^−/−^* mice

**Figure 2:**
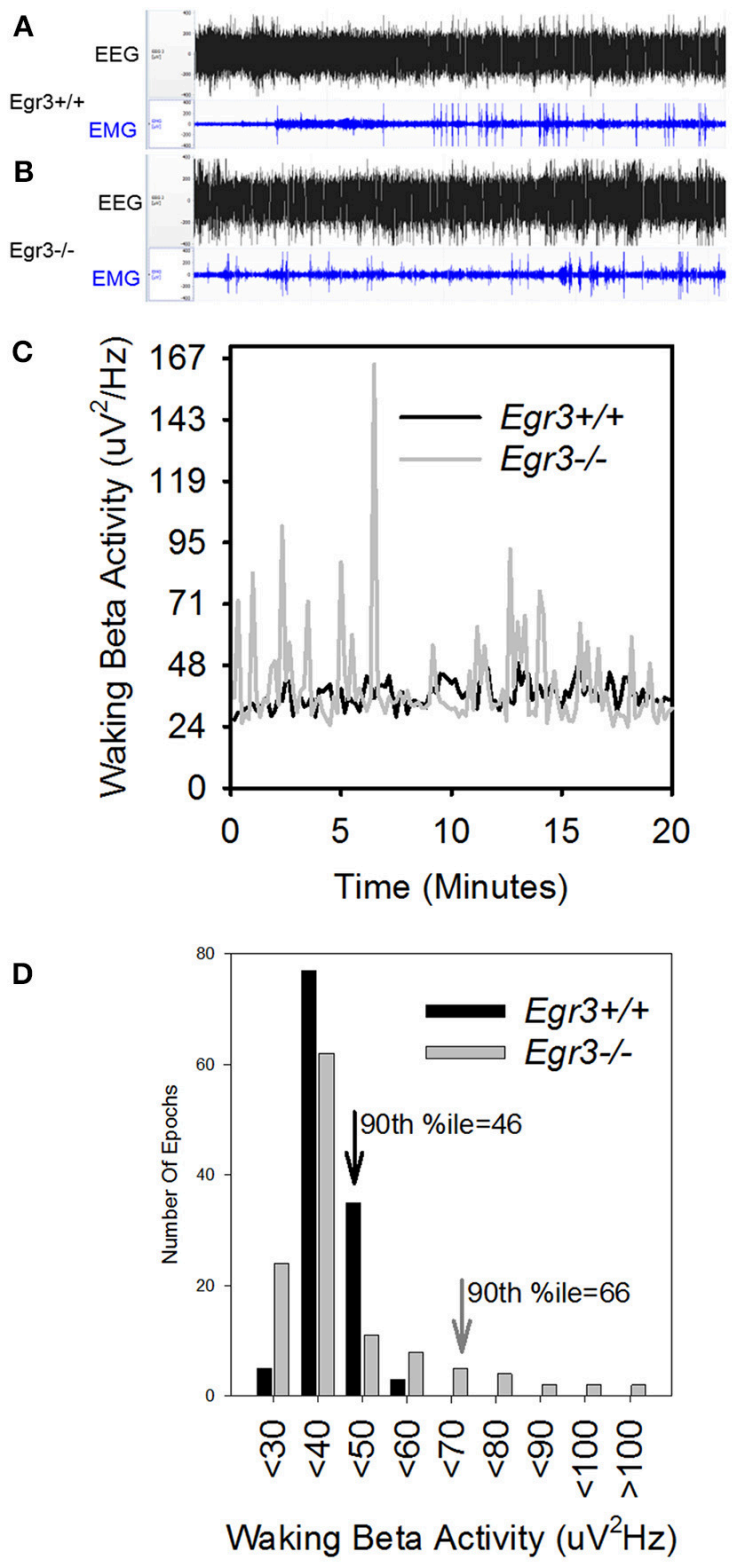
EEG state instability in *Egr3*^−/−^ mice. **(A,B)** EEG (black) and EMG (blue) traces from an *Egr3*^+/+^ **(A)** and an *Egr3*^−/−^ **(B)** mouse, collected during a 20-min interval of wakefulness. **(C)** EEG spectral power in the beta (15–35 Hz) range from a representative *Egr3*^+/+^ (black) and an *Egr3*^−/−^ (gray) mouse during a 20-min interval of wakefulness, plotted in 10 s intervals. **(D)** Data from **(C)** plotted as histograms. The 90th percentile value for spectral power in the beta (15–35 Hz) range from the *Egr3*^+/+^ animal is indicated by a downward pointing black arrow and that of the *Egr3*^−/−^ by a gray arrow.

During 24 h spontaneous wakefulness, the total EEG power in the 1–20 Hz range did not differ between *Egr3*^−/−^ and *Egr3*^+/+^ mice [genotype: *F*_(1, 27)_ = 0.7, *P* = 0.42]. However, the *Egr3*^−/−^ mice exhibited irregular surges in EEG amplitude typical for sleep onset, a surge that did not coincide with EMG quiescence, which is a behavioral marker for sleep onset (Figures 2A–C). Figure 2D illustrates that the modal EEG beta (15–35 Hz) activity did not differ between *Egr3*^−/−^ and *Egr3*^+/+^ mice, approximating 40–50 μV/Hz in both groups. Yet, transient surges of beta power were more frequent and of greater magnitude in *Egr3*^−/−^ mice (mean 43.24 μV/Hz, ranging from 24.92 to 122.03 μV/Hz) compared to *Egr3*^+/+^ mice (mean 32.86 μV/Hz, ranging from 24.25 to 56.69 μV/Hz) (*t* = −9.64, *p* < 0.001; Figure 2C). The fluctuations of waking spectral power in the beta range were highly affected by genotype (*P* < 0.001, η^2^ = 0.40), whereas delta and theta activity did not differ by genotype (delta 1–4 Hz, n.s., η^2^ = 0.02; theta 5–8 Hz, n.s., η^2^ = 0.03; Table 1). Alpha activity was significantly impacted by genotype (*P* = 0.037; Table 1) however, the effect size of genotype for alpha activity (η^2^ = 0.16; Table 1) was considerably smaller than for beta activity. Genotype-dependent epoch-by-epoch EEG power fluctuations in the delta, theta and beta ranges were modulated as a function of vigilance state (Table 1); all were elevated in in *Egr3*^−/−^ mice compared to *Egr3*^+/+^ mice. As in the genotype main effect, the effect size for genotype × state was highest for EEG power in the beta range. Consequently, additional analyses of EEG spectral content were restricted to the beta range.

**Table 1:** Effect of genotype and genotype × state interaction on epoch-by-epoch variation in EEG spectral power.

		**Genotype effect (mean)**			**Genotype effect (90th Pctile)**			**Genotype × state (90th Pctile)**		
**Band**	**Range (Hz)**	***F*_(1, 27)_**	***P***	**Partial η^2^**	***F*_(1, 27)_**	***P***	**Partial η^2^**	***F*_(4, 108)_**	***P***	**Partial η^2^**
Delta	1–4	<0.01	N.S	<0.01	0.56	N.S	0.02	3.85	0.006	0.12
Theta	5–8	1.55	N.S	0.06	0.67	N.S	0.03	4.30	0.003	0.15
Alpha	8–12	8.51	0.007	0.25	4.85	0.037	0.16	1.90	N.S	0.07
Beta	15–35	11.76	0.002	0.32	16.97	<0.001	0.46	11.21	<0.001	0.31

**Figure 3:**
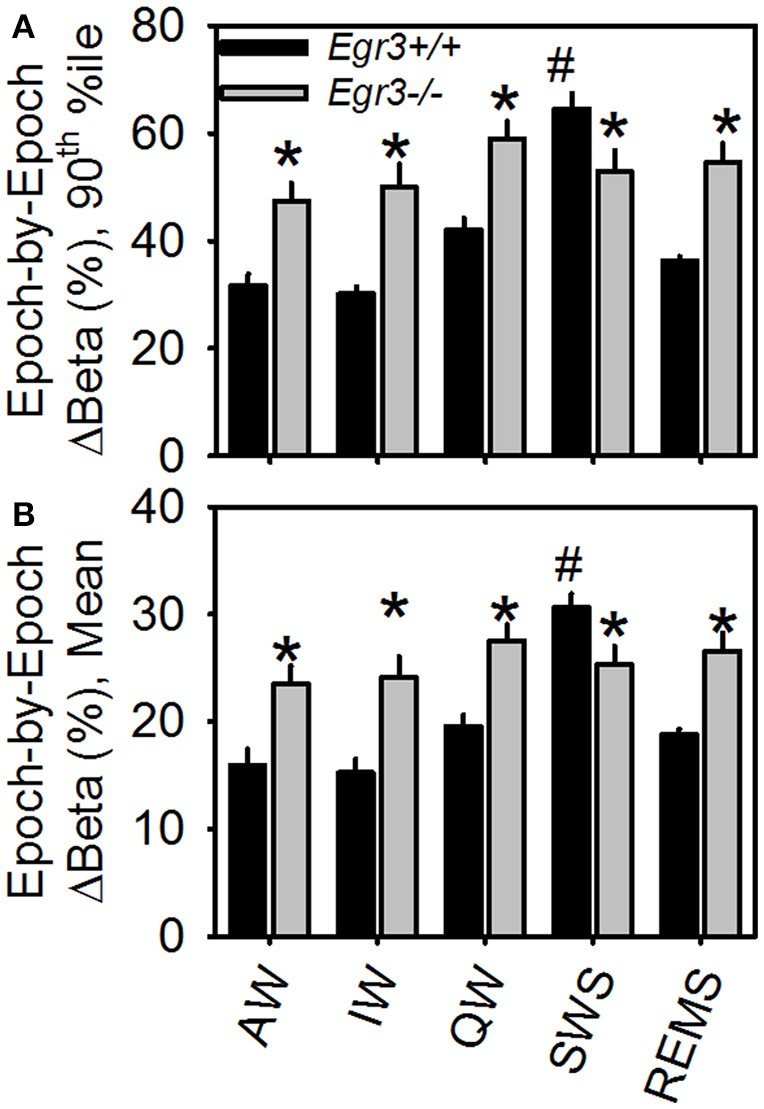
Epoch-by-epoch changes in spectral power in the beta (15–35 Hz) range within each of the five vigilance states. **(A)** 90th percentile values. **(B)** Mean values. *Egr3 ^−/−^ significantly different from *Egr3*^+/+^ within the same vigilance state, Fisher's LSD. ^#^Significantly greater than values from all other states within the same genotype group (*Egr3*^+/+^), Fisher's LSD.

Genotype × vigilance state interaction was significant for both mean epoch-by-epoch change [*F*_(4, 108)_ = 10.9, *P* < 0.001] and 90th percentile epoch-by-epoch change [*F*_(4, 108)_ = 11.2, *P* < 0.001] in beta activity. Vigilance states of AW, IW, QW, and REMS showed greater beta activity instability in *Egr3*^−/−^ mice than *Egr3*^+/+^ mice (Figure 3). Only when the animals were in the state of SWS was the instability of beta activity of lower magnitude in *Egr3*^−/−^ mice than *Egr3*^+/+^. In *Egr3*^+/+^ mice, the stability of beta activity was state dependent: all desynchronized states (AW, IW, QW, and REMS) were significantly lower than SWS, both measured as 90th percentile values (Figure 3A) or mean values (Figure 3B). By contrast, the epoch-by-epoch instability of beta activity did not exhibit any vigilance state dependency in *Egr3*^−/−^ mice.

### Visual evoked potentials

Sleep disruption (SD; 4 h) resulted in a significant increase in time awake in both genotypes relative to time-of-day matched spontaneous sleep recording [*F*_(1, 6)_ = 231.1, *P* < 0.001]. There were no genotype difference in time spent awake (215 ± 6 min in *Egr3*^+/+^ mice vs. 222 ± 6 min in *Egr3*^−/−^ mice). The remainder of time during SD (<10 min/h, both genotypes) was spent in SWS. Repeated measures ANOVA with waking substate (QW vs. AW) as a within subjects factor and genotype as a between subjects factor was used to determine the effect of SD on beta activity in the waking EEG during SD. ANOVA yielded a significant main effect of substate [*F*_(1, 5)_ = 13.3, *P* = 0.015] on EEG beta power (as a percentage of the baseline value recorded from the same animal) without significant genotype × substate interaction. Beta activity was elevated by 36 ± 23% in QW in hours 3–4 of SD relative to spontaneous sleep and not elevated above baseline (13 ± 17%) in AW.

In the 2 h interval following SD, the time spent in wakefulness and SWS was not different from time-of-day matched spontaneous sleep recording (*P* > 0.25). *Egr3*^+/+^ mice spent 54 ± 6 min awake and 53 ± 7 min in SWS following SD. *Egr3*^−/−^ mice spent 45 ± 4 min awake and 65 ± 4 min in SWS. Delta activity in SWS was elevated after SD [SD vs. spontaneous sleep; *F*_(1, 6)_ = 6.9, *P* = 0.040], but not modulated as a function of genotype. During hours 5–6 of recording, delta power in the SWS EEG was elevated by 23% in *Egr3*^−/−^ mice and 19% in *Egr3*^+/+^ mice. These data demonstrate accumulation of sleep need as a consequence of sleep disruption. Time spent in REMS was reduced in *Egr3*^−/−^ mice (9 ± 1 min) relative to *Egr3*^+/+^ (13 ± 2 min) [genotype: *F*_(1, 6)_ = 8.8, *P* = 0.025]. In line with previous findings (Grønli et al., 2016a), time spent in REMS during the entirety of the 6 hrs spontaneous sleep session was reduced in *Egr3*^−/−^ mice (22 ± 6 min) relative to *Egr3*^+/+^ mice [42 ± 4 min; genotype: *F*_(1, 6)_ = 9.2, *P* = 0.023].

**Figure 4:**
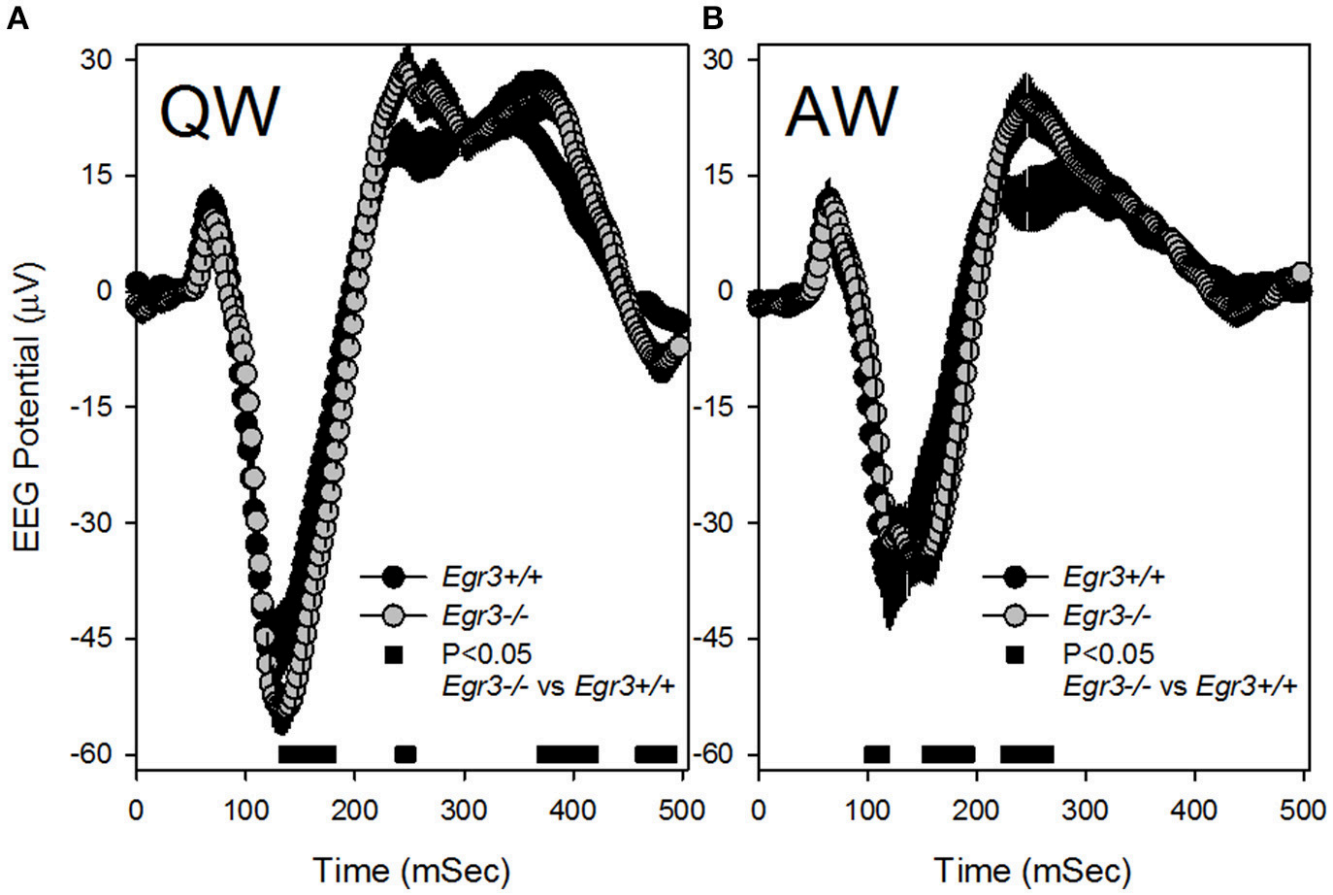
Visual evoked potential (VEP) responses in the first 500 ms after each 10 s blue light exposure (time 0–10 ms on the x-axis). **(A)** VEP responses to stimuli delivered in QW. **(B)** VEP responses to stimuli delivered in AW. Black symbols denote VEP curves from *Egr3*^+/+^ mice and gray symbols denote VEP curves from *Egr3*^−/−^ mice. Black bars at the base of each graph indicate time points at which *post-hoc* comparison with Fisher's LSD indicated a significant difference between genotypes.

Because SWS occurred infrequently during SD and REMS did not occur at all during SD, analysis of VEPs during the SD sessions was restricted to those occurring in wake. Additionally, because the previously observed tonic waking EEG phenotype of *Egr3*^−/−^ mice was manifested in QW selectively (Grønli et al., 2016a), VEPs were sorted for waking substate (AW vs. QW). VEPs consisted of a negative deflection of the EEG that peaked 150 msec after stimulus onset, followed by a modest and broader positive deflection peaking approximately 250 ms after stimulus onset (Figure 4). VEP waveforms varied as a function of waking substate [QW vs. AW: *F*_(48, 672)_ = 49.4, *P* < 0.001]. Both the negative and positive VEP peaks were of higher magnitude during QW (Figure 4A) relative to AW (Figure 4B). The effect of waking substate on VEP waveforms also varied as a function of genotype [genotype × substate × time: *F*_(48, 672)_ = 7.0, *P* < 0.001]. For light pulses delivered in QW*, Egr3*^−/−^ mice responded with a significantly more negative potential from 135 to 175 msec after stimulus onset, relative to *Egr3*^+/+^ mice, and a more positive potential in intervals between 200 and 450 ms after stimulus onset (Figure 4A). For light pulses delivered in AW, a significantly more negative potential was reached in *Egr3*^−/−^ mice, relative to *Egr3*^+/+^ mice, from 157 to 190 msec after stimulus onset, and a more positive potential in the interval between 200 and 300 ms after stimulus onset, compared to *Egr3*^+/+^ mice (Figure 4B).

**Figure 5:**
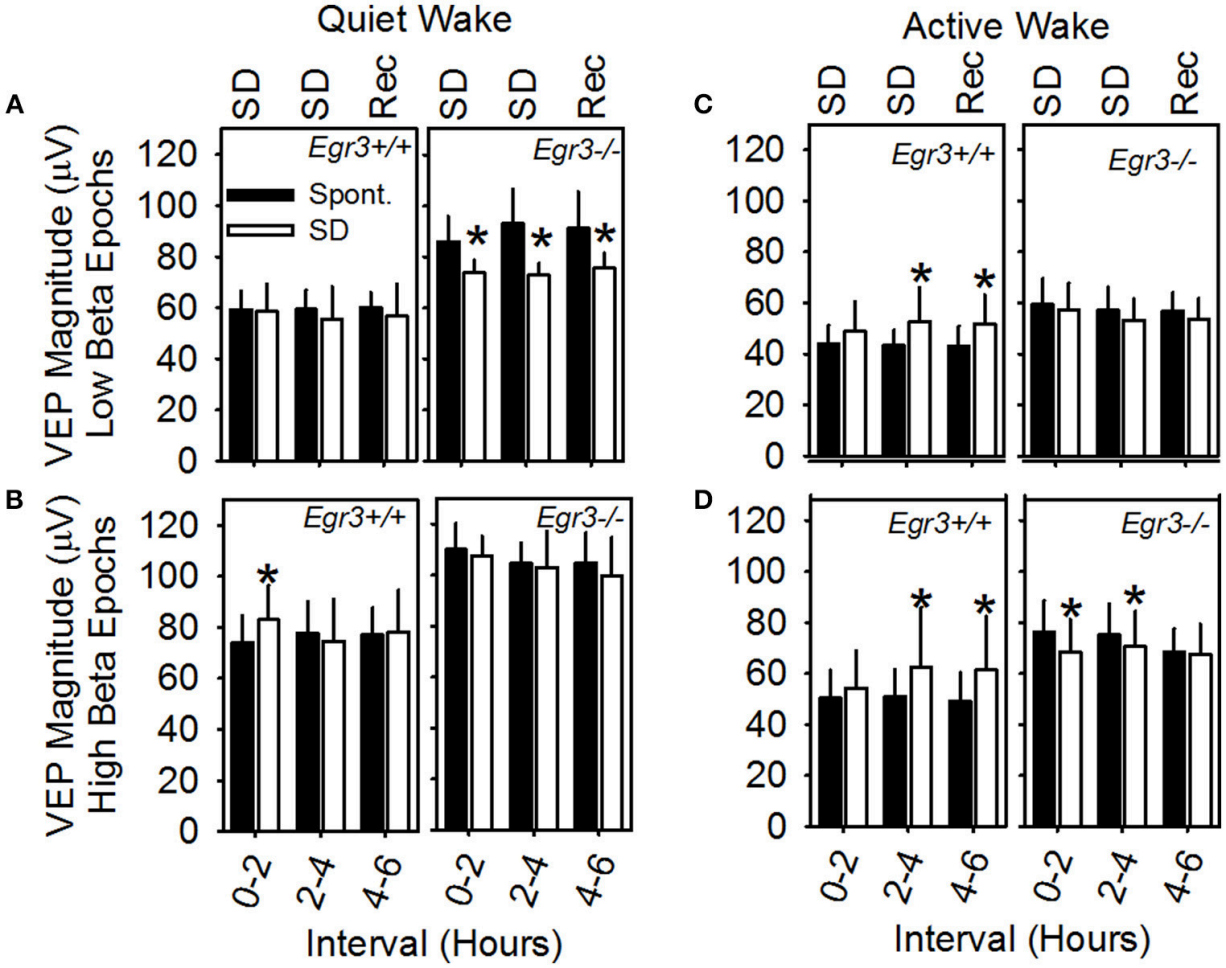
VEP magnitude as a function of waking substate and EEG spectral power in the beta (15–35 Hz) range. Data are from **(A)** QW epochs with low amounts of EEG beta activity, **(B)** QW epochs with high amounts of EEG beta activity, **(C)** AW epochs with low amounts of EEG beta activity, **(D)** AW epochs with high amounts of EEG beta activity. Text at the top of A and C panels indicates the experimental condition from which data represented by white bars are derived: SD, sleep disruption; Rec, recovery; post-SD. Data from the equivalent 2 h intervals on the baseline day are represented by black bars (Spont., spontaneous sleep). *Significantly different from baseline/spontaneous sleep day in the same genotype group and analysis interval, Fisher's LSD.

A mixed-design ANOVA was applied (between factor genotype and within factors waking substate (AW vs. QW), beta activity (high vs. low), day (SD vs. spontaneous sleep) and interval (hours 1–2 vs. hours 3–4 vs. hours 5–6), with peak-to-peak VEP magnitude as the dependent variable (Figure 5). Waking substate exhibited the largest main effect size [*F*_(1, 6)_ = 197.7*, P* < 0.001; partial η^2^s = 0.97], as well as significant interactions: substate X genotype [*F*_(1, 6)_ = 15.3, *P* = 0.008] and substate × day × interval [*F*_(2, 12)_ = 5.4, *P* = 0.020]. QW exhibited an elevated VEP magnitude relative to active wakefulness (Figures 5A,B vs. Figures 5C,D). This effect was exaggerated in *Egr3*^−/−^ mice relative to *Egr3*^+/+^ mice. Irrespective of other within-subjects variables, VEP magnitude was elevated by 47% during QW relative to AW in *Egr3*^−/−^ mice and only 33% during QW relative to AW in *Egr3*^+/+^ mice. Although the genotype effect on VEP magnitude was not significant in any single interval when correcting for multiple comparisons (*P* ≥ 0.025, *post-hoc* comparison), VEP magnitude measured in QW was elevated in *Egr3*^−/−^ mice relative to *Egr3*^+/+^ mice by more than 25% consistently across days and times (Figures 5A,B). By contrast, VEP magnitude measured in AW was elevated in *Egr3*^−/−^ mice relative to *Egr3*^+/+^ mice by as little as 1% (Figures 5C,D). This phenotype, like the originally reported intrusion of slow activity into the EEG, is thus manifested state-specifically in QW.

Independently of genotype, the occurrence of beta activity in the EEG exhibited a main effect on VEP magnitude [*F*_(1, 6)_ = 41.0, *P* < 0.001; partial η^2^s = 0.87] but no interaction with substate. Irrespective of genotype, VEP magnitude was 23% greater in AW epochs characterized by high beta activity (Figure 5D) than in AW characterized by low beta activity (Figure 5C). It was 26% greater in QW epochs characterized by high beta activity (Figure 5B) than in QW characterized by low beta activity (Figure 5A).

The significant interaction of substate × day × interval [*F*_(2, 12)_ = 5.4, *P* = 0.020] reflects the modulation of VEP amplitude as a consequence of SD. However, when significant in *post hoc* comparisons, changes in VEP magnitude on the SD day relative to spontaneous sleep were distinct across genotypes (Figure 5): whereas *Egr3*^−/−^ mice consistently exhibited a reduction in VEP amplitude during and after SD relative to the baseline day, *Egr3*+ mice exhibited an increase in VEP amplitude during SD relative to the baseline day.

**Figure 6:**
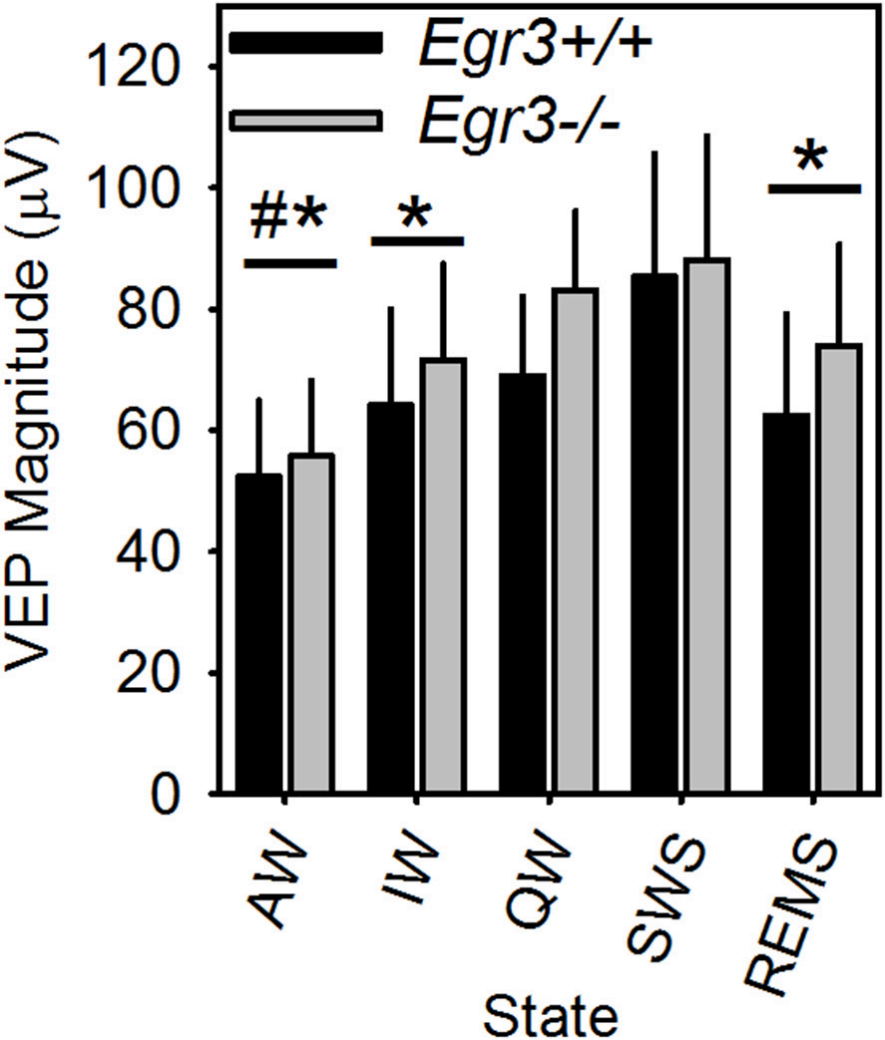
VEP magnitude varies as a function of vigilance state in *Egr3*^+/+^ (black bars) and *Egr3*^−/−^ (gray bars) mice. *Significantly lower VEP magnitude relative to SWS (irrespective of genotype; Fisher's LSD). ^#^Significantly lower VEP magnitude relative to QW (irrespective of genotype; Fisher's LSD).

Occurrence of all vigilance states, including REMS, in the 2-h post-SD interval allowed for comparison of peak-to-peak VEP magnitude across all vigilance states in this interval. VEP magnitude differed across vigilance states [*F*_(4, 24)_ = 10.5, *P* < 0.001; Figure 6]. VEP magnitude was higher in SWS and QW in comparison to AW, and higher in SWS compared to IW and REMS. VEP magnitude did not differ between SWS and QW nor across AW, IW, and REMS. When considered across all vigilance states, VEP magnitude was not significantly modulated as a function of genotype.

## Discussion

Wakefulness is defined polysomnographically by relatively low amplitude oscillatory activity in the EEG relative to SWS, though higher amplitude events intrude into the EEG transiently during QW as a result of accumulated sleep need (Grønli et al., 2016b). This report demonstrates that vigilance substate-specific EEG events are paralleled by modulation of VEPs within wakefulness. The VEP responses were significantly larger during QW than AW and equivalent in magnitude to those that occurred in SWS. Moreover, we found that the VEP magnitude was higher in those epochs characterized with higher beta activity, an EEG marker previously linked to the accumulation of sleep need over time (Grønli et al., 2016b). Mice lacking the plasticity-regulatory immediate early gene *Egr3* display, in conjunction with elevated beta activity, elevated evoked response magnitude resembling that of animals in SWS. Collectively, these observations indicate that *Egr3* is necessary to maintain stability in the arousal state of cerebral cortical networks during wakefulness.

The shape and magnitude of VEPs varied as a function of polysomnographically-defined vigilance states, as has been reported elsewhere for sensory evoked potentials in both animals (Rector et al., 2005, 2009; Phillips et al., 2011) and humans (Weitzman and Kremen, 1965; Nielsen-Bohlman et al., 1991). Also, as described previously for auditory evoked potentials in humans in QW prior to sleep onset (Weitzman and Kremen, 1965), VEP is elevated in QW relative to AW. The state-dependence of VEP magnitude has been theoretically attributed to an elevated driving potential when the animal transitions from AW to QW to SWS in response to increasing sleep drive. Thalamocortical and cortical cells involved in generating VEPs undergo hyperpolarization as a consequence of the loss of ascending subcortical depolarizing neuromodulatory tone (Crunelli and Hughes, 2010; David et al., 2013). When the visual stimulus is delivered, the magnitude of the resulting depolarization will be greater when initiated from the relatively hyperpolarized state occurring in SWS than from the relatively depolarized state occurring in active wakefulness (Phillips et al., 2011).

The data introduced in the current report expands on the previous observation of elevated EEG markers for sleep need in the EEG of the *Egr3*^−/−^ mouse (Grønli et al., 2016a), by demonstrating a significant interaction of genotype and state in affecting delta, theta, alpha and beta activities (Table 1). It is important to consider the interaction of genotype × state specifically, because in wild type mice sleep need markers in the EEG are detected in quiet wake only and obscured in active wake (Grønli et al., 2016a,b). As we demonstrated previously (Grønli et al., 2016b), beta activity in QW is a marker for sleep need that parallels delta activity. Both of these measures increase, during quiet wake specifically, as a function of prior waking duration. But in the current study, the genotype and genotype × state effect sizes on epoch-by-epoch EEG dynamics, were larger for beta activity than delta activity; consequently, the subsequent analysis focused on beta activity. This observation also illustrates the value of beta activity as a high frequency EEG marker for accumulation of sleep need. It can be measured on a smaller time scale than delta activity, and in this capacity beta activity may augment slow wave detection in the waking EEG as a marker for sleepiness.

Beta activity measured in QW tracks the accumulation of sleep need, in that beta activity increases with time spent awake. Because VEP magnitude is elevated in QW epochs characterized by high beta activity relative to those characterized by lower beta activity across epochs of QW (see Figure 5A vs. Figure 5B), it was hypothesized that VEP magnitude might also serve as a marker for accumulation of sleep need over time. However, the current data did not bear out this prediction consistently. The effect of SD in *Egr3*^+/+^ mice was to elevate VEP magnitude in QW and AW (Figures 5B–D) albeit modestly, an effect which resembles the shift in VEP magnitude from AW to QW/SWS and thus may reflect accumulating sleep need. However, the effect of SD in *Egr3*^−/−^ mice was the opposite: suppression of VEP magnitude (Figures 5A,D). *Egr3*^−/−^ mice are hyperreactive to novelty and handling at the behavioral level and in the HPA axis (as measured by blood corticosterone levels) (Gallitano-Mendel et al., 2007). Hyperreactivity to the sensory stimulation associated with SD may have masked any effect of accumulated sleep need on cortical processing of visual information in these mice.

A likely contributor to the genotype effect is the deficit in serotonin 5HT_2a_ signaling that occurs in the cerebral cortex of *Egr3*^−/−^ mice (Williams et al., 2012; Maple et al., 2015). Serotonin is among the ascending subcortical depolarizing neuromodulators that are associated with wakefulness and withdrawn in sleep (Portas et al., 1998; Bjorvatn et al., 2002), and may thus contribute to state-dependent changes in EEG power and VEP shape, and their modulation by *Egr3* genotype. The deficit is apparent in the reduced binding of 5HT_2a_ receptor ligands in cerebral cortical tissue samples in *Egr3*^−/−^ mice (Williams et al., 2012). Moreover, *Egr3*^−/−^ mice fail to respond at behavioral (Williams et al., 2012) and electroencephalographic (Grønli et al., 2016a) levels to 5HT_2a_ receptor antagonists. Since the 5HT_2a_ receptor is known to promote cortical arousal during wake (Popa et al., 2005), and 5HT_2a_ availability, as assessed by positron emission tomography, is elevated in the cerebral cortex of humans during protracted wake (Elmenhorst et al., 2012), we hypothesize that the elevated magnitude of VEPs in *Egr3*^−/−^ mice is a consequence of the loss of 5HT_2a_ tone. Loss of 5HT_2a_ tone reduces cortical connectivity to a state akin to excessive sleepiness. Ultimately, more direct manipulations of 5HT2a signaling within the cerebral cortex will be needed to ascertain the local role of this receptor in regulating manifestations of sleepiness and the concomitant changes in cortical VEP shape.

*Egr3* is an immediate-early gene transcription factor implicated in schizophrenia susceptibility (Yamada et al., 2007; Huentelman et al., 2015), and expressed at reduced levels in post-mortem brain tissue from schizophrenia patients (Dean and Hayes, 1996; Mexal et al., 2005; Yamada et al., 2007). Reduced 5HT_2a_ signaling is hypothesized to underlie deficits in the processing of sensory information, as assessed by prepulse inhibition, in schizophrenics (Maier et al., 2008). Thus, the modulation of both 5HT_2a_ signaling and VEP magnitude in wakefulness as a function of *Egr3* genotype suggest a potential role for *Egr3* as a genetic regulator of abnormalities in experience-dependent processes in schizophrenia.

The VEP, when monitored at the level of field potentials, is an integrative measure of electrical potentials generated at multiple levels in the nervous system. The VEP is generated by the optic nerve, the lateral geniculate bodies of the thalamus, the optic tracts and their radiations to the visual cortex, and the activity of the visual cortex itself (Epstein, 2000). It is therefore not possible to attribute the genotype- or state-specific differences that were observed to genotype- or state-specific electrophysiological differences in any of the above listed structures. There are no gross abnormalities in nervous system structure in *Egr3*^−/−^ mice (Li et al., 2007). These observations and the fact that the genotypic difference in VEP magnitude is largely restricted to QW indicate that the genotypic difference is not a gross abnormality in visual function but an arousal-related abnormality in communication within the nervous system.

Beta activity in the EEG is a reliable covariate of insomnia in humans, one that has led to the speculation that it indicates hyperarousal (Merica et al., 1998; Perlis et al., 2001; Wassing et al., 2016). Yet in experimental rodents, it tracks cumulative sleep drive during protracted wake (Grønli et al., 2016b). As shown here, in comparisons across epochs and controlling for state, elevated beta activity coincides with elevated VEP magnitude, an indication of decreased cortical arousal, not increased cortical arousal. The work in rodents leads to the conclusion that in fact beta activity is a marker for hypersomnolence, and that elevated beta activity in insomniacs may indicate elevated sleep need due to the prior failure to discharge sleep need. Additional work in humans will be necessary to ascertain the generalizability of the relationship between beta activity and sleep need.

## Conclusion

In summary, an EEG feature that tracks accumulated sleep need over time, elevated beta activity in the quiet waking EEG, has been shown here to be accompanied by an elevation of VEP magnitude resembling that occurring in sleep. These two phenomena have been shown here to be regulated genetically by the transcriptional regulatory immediate early gene *Egr3*, the expression of which is known to vary as a function of sleep state (Terao et al., 2003; Maple et al., 2015). Druggable downstream targets of *Egr3*, including the 5HT_2a_ receptor, may prove useful in combatting sleepiness or insomnia. Functional polymorphisms in *Egr3* and its regulatory targets such as *Arc* may prove useful biomarkers for sensitivity to the negative impacts of sleepiness on cognition, as they have for symptoms of schizophrenia risk (Huentelman et al., 2015). Related schizophrenia endophenotypes, including loss of prepulse inhibition that resemble, at least phenomenologically, those brought on by sleep deprivation (Ettinger and Kumari, 2015) support the concept that the neurobiological underpinnings of schizophrenia are in part subject to modulation by sleep/wake regulatory mechanisms. *Egr3* may be the molecular switch that mediates this relationship.

## Author contributions

JG, MS, and JW planned experiments. MS performed all experimental procedures and manipulations and collected all data. MS and JW analyzed data. JW performed statistical analyses. JG, MS, and JW interpreted results. JG, MS, and JW wrote portions of the manuscript.

### Conflict of interest statement

The authors declare that the research was conducted in the absence of any commercial or financial relationships that could be construed as a potential conflict of interest.
